# Early Feeding Practices and Celiac Disease Prevention: Protocol for an Updated and Revised Systematic Review and Meta-Analysis

**DOI:** 10.3390/nu14051040

**Published:** 2022-02-28

**Authors:** Hania Szajewska, Raanan Shamir, Anna Chmielewska, Agata Stróżyk, Bartłomiej M. Zalewski, Renata Auricchio, Sibylle Koletzko, Ilma R. Korponay-Szabo, Luisa Mearin, Caroline Meijer, Carmen Ribes-Koninckx, Riccardo Troncone, on behalf of the PREVENTCD Study Group

**Affiliations:** 1 Department of Paediatrics, The Medical University of Warsaw, 02-091 Warsaw, Poland; ag.strozyk@gmail.com (A.S.); zalewski.bm@gmail.com (B.M.Z.); 2 Schneider Children’s Medical Center of Israel, Institute of Gastroenterology, Nutrition and Liver Diseases, Petach Tikva 4920235, Israel; raanan@shamirmd.com; 3 Department of Clinical Sciences, Pediatrics, Umeå University, 901 85 Umeå, Sweden; anna.chmielewska@umu.se; 4 Pediatric Section European Laboratory for the Investigation of Food Induced Disease (ELFID), Department of Translation Medical Science, University Federico II, 80331 Naples, Italy; r.auricchio@unina.it (R.A.); troncone@unina.it (R.T.); 5 Department of Pediatrics, Dr. von Hauner Children’s Hospital, University Hospital, LMU Munich, 80337 Munich, Germany; sibylle.koletzko@med.uni-muenchen.de; 6 Department of Pediatrics, Gastroenterology and Nutrition, School of Medicine Collegium Medicum, University of Warmia and Mazury, 11082 Olsztyn, Poland; 7 Department of Pediatrics, Faculty of Medicine and Clinical Center, University of Debrecen, 4032 Debrecen, Hungary; ilma.korponay-szabo@tuni.fi; 8 Celiac Disease Center, Heim Pál Children’s Hospital, 1089 Budapest, Hungary; 9 Department of Pediatrics, Leiden University Medical Center, Willem Alexander Children’s Hospital, 2300 RC Leiden, The Netherlands; M.L.Mearin_Manrique@lumc.nl (L.M.); c.r.meijer-boekel@lumc.nl (C.M.); 10 Pediatric Gastroenterology and Hepatology, La Fe University Hospital, 46026 Valencia, Spain; ribes_car@gva.es

**Keywords:** nutrition, feeding, infants, children, celiac sprue, breastfeeding

## Abstract

Uncertainty remains in regard to when, how, and in what form gluten should be introduced into the diet, particularly of infants genetically predisposed to developing celiac disease (CD). MEDLINE (PubMed), EMBASE, and Cochrane Central Register of Controlled Trials databases will be searched from inception. Randomized controlled trials (RCTs) and observational studies (cohort, case-control, or cross-sectional studies) investigating the association between early feeding practices and the risk of CD and/or CD autoimmunity will be included. In prospective studies, participants will be infants regardless of the risk of developing CD. For retrospective studies, participants will be children or adults with CD or presenting with positive serology indicative of CD. Interventions will be gluten-containing products of any type. Exposures will be breastfeeding and/or the introduction of gluten-containing products of any type. In control groups, there will be no exposure, different degrees of exposure (partial vs. exclusive breastfeeding, different amounts of gluten, etc.), or a placebo. The primary outcome measure will be CD or CD autoimmunity (i.e., anti-transglutaminase or anti-endomysial antibodies). At least two reviewers will independently assess the risk of bias using a validated risk assessment tool depending on study design. Disagreements will be resolved by discussion to achieve a consensus with the involvement of one or more additional reviewers if required. If appropriate, data will be pooled. If not, a narrative synthesis will be performed. The findings will be submitted to a peer-reviewed journal.

## 1. Introduction

Celiac disease (CD) is “an immune-mediated systemic disorder elicited by gluten and related prolamines in genetically susceptible individuals and characterized by the presence of a variable combination of gluten-dependent clinical manifestations, CD specific antibodies, HLA-DQ2 or HLA-DQ8 haplotypes, and enteropathy” [1,2]. The prevalence of positive CD serology can be in as high as 8.5% of the population, although overall CD prevalence is 1.4% based on serologic tests and 0.7% based on biopsy [3]. Recent evidence suggests that the incidence and prevalence of CD are rising in the pediatric age group [4,5,6].

Given the high prevalence and health burden of CD due to morbidity associated with the disease and because it is a permanent condition when developed [7,8,9,10], preventive strategies targeted at reducing the occurrence of CD should be considered a priority. Since gluten is a known trigger for eliciting an abnormal immune response in a portion of genetically predisposed individuals, the introduction of gluten into infants’ diet, along with the effects of breastfeeding, has been extensively studied. In line with 2016 recommendations on gluten introduction and risk of CD by the European Society for Pediatric Gastroenterology, Hepatology and Nutrition (ESPGHAN) [11], gluten can be introduced into infants’ diet between the ages of 4 and 12 completed months. Neither any breastfeeding nor breastfeeding at the time of gluten introduction can be recommended as a means of reducing the risk of CD. No recommendation was made regarding the type of gluten to be used at introduction. Based on limited evidence, it was suggested that the consumption of large amounts of gluten should be discouraged during the first months after gluten introduction. Because of a lack of consensus within the group on how to interpret the limited evidence available, no recommendation was made on gluten introduction in children with first-degree relatives with CD.

Since the publication of the ESPGHAN guidelines, new evidence has emerged, further suggesting that the association between breastfeeding and the amount of gluten at introduction matters [12,13,14]. Furthermore, more information has become available on the relationship between genetic risk and gluten introduction [12,15]. Therefore, an updated systematic search of literature is being undertaken by the members of the Prevent CD group, which assisted in formulating the ESPGHAN recommendations.

The main objective of this review is to systematically revise and update a 2015 systematic review and meta-analysis on early feeding practices and the risk of celiac disease [16]. The intention is that this updated meta-analysis will serve as a basis for revising the 2016 ESPGHAN guidelines [11] on gluten introduction and the risk of CD and CD autoimmunity. As previously, our systematic review is designed to answer the following clinical questions:

- Breastfeeding and CD. Is the risk of developing CD reduced by exclusive or any breastfeeding? Is CD development age influenced by exclusive or any breastfeeding? Is the risk of developing CD affected by breastfeeding duration?
- Breastfeeding at the time of gluten introduction and CD.Is the risk of CD reduced if gluten is consumed during breastfeeding?
- Timing of gluten introduction: Is the risk of developing CD influenced by the timing of gluten introduction? Does the age of gluten introduction affect the age when CD develops?
- Amount of gluten at weaning (and later) and CD. Is the amount of gluten consumed an independent risk factor for CD development in early childhood? Is there a threshold level of gluten consumption for this risk?
- Type of gluten: Is CD risk influenced by the type of cereal (wheat, rye, barley) consumed at gluten introduction or later during childhood? Does the type of gluten-containing products (bread, porridge, follow-on formula) at gluten introduction influence CD risk?
- Gluten during lactation. Is CD risk in the offspring influenced by implementation of a gluten-free diet vs. a gluten-containing diet during lactation?
- Genetic predisposition. Does the risk of developing CD differ between low- and high-risk populations (“genetic load”)?

## 2. Materials and Methods

### 2.1. Reporting Guidelines

We will perform a systematic review and meta-analysis using the guidelines from the Cochrane Collaboration for undertaking and reporting the results of a systematic review and meta-analysis [17]. The protocol is reported according to the Preferred Reporting Items for Systematic Review and Meta-Analysis Protocols guideline (PRISMA), and the findings will be reported in line with the PRISMA guidelines [18]. The study protocol was registered at PROSPERO (CRD42021248583).

### 2.2. Criteria for Considering Studies for this Review

#### 2.2.1. Types of Studies

We will include all randomized controlled trials (RCTs) and observational (cohort, case-control, or cross-sectional) studies investigating the potential association between early feeding practices and the risk of CD and/or CD autoimmunity. Animal studies, case reports/case-series reports, editorials, or letters to the editor will be excluded. Studies assessing the association of early feeding practices only with the age of CD development (delay of onset) will be included.

#### 2.2.2. Types of Participants

In prospective studies, participants will be infants regardless of the risk of developing CD. Studies on unselected infants from the general population and on infants with known genetic risk for CD (HLA DQ2 or DQ8) will be assessed separately. For retrospective studies, participants will be children or adults with CD (diagnosed by small bowel biopsy or according to an appropriately applied biopsy-sparing protocol based on ESPGHAN guidelines) or those presenting with positive serology indicative of CD, i.e., anti-transglutaminase antibody (TGA) or endomysial antibody (EMA) positivity. Participants with CD diagnosed by deamidated gliadin peptide (DGP) or conventional gliadin antibodies only will be excluded. In the case of an updated version of study results (including the same cohort of participants), only the most recent publication (follow-up) will be included. 

#### 2.2.3. Types of Interventions

Interventions eligible for inclusion will be gluten-containing products of any type (such as cereals, flour, or any other foods containing gluten or its homologues or mimotopes or preparations manufactured for research purposes). Exposures eligible for assessment will be breastfeeding and/or the introduction of gluten-containing products of any type. In control groups, there will be no exposure, different degrees of exposure (partial vs. exclusive breastfeeding, different amounts of gluten, etc.), or a placebo.

#### 2.2.4. Types of Outcome Measures

The primary outcome measure is CD or the development of CD autoimmunity (i.e., TGA or EMA).

#### 2.2.5. Search Methods for the Identification of Studies

Search will be undertaken for studies from inception to November 2021 in the following electronic databases: MEDLINE (PubMed), EMBASE, and the Cochrane Central Register of Controlled Trials (CENTRAL, the Cochrane Library) databases. Researchers working in the field will be contacted for any additional, unpublished studies. Previously published systematic reviews will be screened for relevant citations. Letters to the editor, abstracts, and proceedings from scientific meetings will be excluded. No language restriction will be imposed. Studies will be included regardless of study duration and length of follow-up. The ClinicalTrials.gov and ClinicalTrialsRegister.eu websites and the International Clinical Trials Registry Platform (ICTRP, https://trialsearch.who.int/) will be also searched for studies that were registered but have not yet been published.

The search strategy was developed by an independent methodologist and reviewed by the review authors (AS/FB/BMZ/AC/HS/RS). The search will be performed independently by four reviewers (AS/AC/BMZ/FB). The title, abstract, and keywords of every study identified with the search strategy and other sources will be screened using the Covidence (https://www.covidence.org/) and EndNote X9 Computer program (Version 9.3.3. Philadelphia, The Clarivate Analytics, 2020). For an example of the search strategy, see Supplementary File S1.

### 2.3. Data Collection and Analysis

#### 2.3.1. Selection of Studies and Data Extraction

Initially, two pairs of reviewers (AS/FB and AC/BMZ) will independently screen titles, abstracts, and keywords of every record identified and code them as “retrieve” (eligible or potentially eligible/unclear) or “do not retrieve.” If both pairs exclude the record, it will be eliminated from further review. If at least one pair of the reviewers includes the records, then full texts of the studies considered as relevant will be obtained. The same pairs of reviewers will independently assess the eligibility of each potentially relevant study against the eligibility criteria. Any disagreement will be resolved through discussion, or, if required, other members of the core review team (HS/RS) will be consulted. The reviewers will extract data from the included studies independently using a standardized data extraction form for study characteristics, outcome data, and quality. The following data will be extracted from each included study:

- Study baseline data: Reference, study design, duration of study, sample size, number of participants lost to follow-up/withdrawn, number of participants analyzed, and study setting.
- Participants: Mean age, inclusion criteria, and exclusion criteria.
- Type of intervention: Duration, frequency, and dosage of the intervention (e.g., recorded by food frequency questionnaires, diet records, and 24 h recall, if available) and type of comparison.
- Types of outcomes: Primary and secondary outcomes and reported time points;
- For non-RCTs, adjusted and unadjusted outcome measures.
- Confounders: Uncontrolled confounders and a list of all confounders for which adjustments have been made. The following confounders have been defined a priori to be relevant to interfere with the primary outcomes: socioeconomic status, maternal education, gestational age, birth weight, birth order and gender, mode of delivery, family history of celiac disease, and diet of the mother related to gluten intake.
- Funding and sponsorship.
- Conflicts of interest.

#### 2.3.2. Assessment of Risk of Bias in Included Studies

The risk of bias in the included studies will be assessed independently by the pairs of reviewers with no blinding with respect to the authors or the journal. The second version of the Cochrane Collaboration’s tool for assessing the risk of bias (ROB-2) will be used for RCTs, as recommended in The Cochrane Collaboration Handbook [17]. This tool evaluates potential sources of bias in five domains: bias introduced by the randomization process, bias caused by deviations from intended interventions, bias due to missing outcome data, bias in outcome measurement, and bias in choosing the reported result. For each individual domain, the trials can be classified into low risk of bias (judgment that the study is at low risk of bias for all domains for this result), some concerns (judgment that the study is at some risk of bias in at least one domain for this result), and high risk of bias (judgment that the study is at high risk of bias in at least one domain for this result or that the study raises some concerns for multiple domains in a manner that significantly reduces confidence in the result).

For observational studies (i.e., cohort, case-control, and cross-sectional studies), the risk of bias will be assessed with the use of the Newcastle–Ottawa Scale (NOS) (including the adapted version for cross-sectional studies) (see Supplementary File S2) [19]. This tool evaluates study quality in three domains: selection of participants, comparability of study groups, and the assessment of outcomes (or ascertainment of exposure in the case of case-control studies). For assessment of each domain, this scale uses a star system (with a maximum of 9 stars for cohort and case-control studies and a maximum of 10 stars for cross-sectional studies). Any disagreement among reviewers will be resolved through discussion, or, if required, other members of the core review team (HS, RS) will be consulted.

#### 2.3.3. Measure of Treatment Effect

The dichotomous outcomes, the results for individual studies, and pooled statistics will be reported as the risk ratio (RR) or the odds ratio (OR) between the experimental and control groups with 95% confidence intervals (95% CIs). The continuous outcomes will be reported as the mean difference (MD) between the treatment and control groups with 95% CI.

#### 2.3.4. Dealing with Missing Outcome Data

If possible, missing outcome data such as standard deviation will be calculated from the available data (*p*-values, *t*-values, CI, or standard errors) using formulas recommended in The Cochrane Handbook [17].

#### 2.3.5. Assessment of Heterogeneity

Heterogeneity will be quantified by *X*^2^ and *I*^2^, which can be interpreted as the percentage of the total variation between studies that is attributable to heterogeneity rather than to chance. A value of 0% indicates no observed heterogeneity, and larger values show increasing heterogeneity. All analyses will be based on the random-effects model.

#### 2.3.6. Assessment of Reporting Biases

When at least 10 RCTs are available, publication bias will be assessed using the funnel plot proposed by Egger et al. [20]. A *p*-value less than 0.05 implies publication bias.

#### 2.3.7. Data Synthesis

The decision on data pooling will be assessed separately for different study designs (interventional and observational studies). Whether data pooling is appropriate will be decided based on the degree of clinical and statistical heterogeneity between studies.

If appropriate, the data will be analyzed the Review Manager (RevMan) computer program. In the case of large heterogeneity, a narrative synthesis will be performed. Depending on the original publication, the binary measure for individual studies will be reported as the risk ratio (RR) or as the odds ratio (OR) between the experimental and control groups or as the hazard ratio (HR), both with a 95% CI. Continuous outcomes will be given as the mean with the standard deviation or the median with ranges. If possible, the mean difference (MD) between treatment and control groups will be selected to represent the difference in continuous outcomes (with a 95% CI). If feasible, the certainty of evidence will be assessed using the grading of recommendations, assessment, development, and evaluation (GRADE) methodology [21].

#### 2.3.8. Subgroup Analysis

Subgroup analyses according to the risk of CD will be performed. Studies on infants at low risk of CD (general population) and at increased risk of CD (defined by the HLA-DQ2/DQ8 status, a first-degree relative with CD, or a disease associated with CD, such as type 1 diabetes mellitus) will be assessed separately.

## 3. Ethics and Dissemination

### 3.1. Ethics

Ethical approval is not needed.

### 3.2. Dissemination

The findings of this review will be submitted to a peer-reviewed scientific journal (pediatric, nutrition, or gastroenterology). Abstracts will be submitted to relevant national and international conferences. 

### 3.3. Patient and Public Involvement

There was no patient or public involvement in the development of this systematic review protocol. 

### 3.4. Limitations

We acknowledge several limitations of the proposed review. First, only a limited number of RCTs may be available. Observational studies will be included in our review. These studies reflect real-life clinical practice and include a broader range of participants with longer follow-up. However, they will identify associations only, not causality. Additional research will be needed to understand whether a causal relationship exists. Moreover, observational studies are likely subject to confounding bias defined as “a distortion that modifies an association between an exposure and an outcome, caused by the presence of an indication for the exposure that is the true cause of the outcome” [22]. The confounding bias may substantially compromise the validity of the findings. 

## 4. Conclusions

The role of early feeding practices, especially in high-risk populations, is still under discussion. Our review intends to serve as a basis for revising the guidelines of the ESPGHAN on gluten introduction and the risk of celiac disease, particularly in infants genetically predisposed to developing celiac disease.

## Data Availability

Not applicable.

## Supplementary Materials

The following supporting information can be downloaded at https://www.mdpi.com/article/10.3390/nu14051040/s1: Supplementary File S1: Search strategy, Supplementary File S2: The Newcastle-Ottawa Scale (NOS) (including adapted version for cross-sectional studies).
