# Supplementary material for: Early Feeding Practices and Celiac Disease Prevention: Protocol for an Updated and Revised Systematic Review and Meta-Analysis
Source: Nutrients. 2022 Feb 28;14(5):1040. doi: 10.3390/nu14051040 (PMC8912481; doi:10.3390/nu14051040)
Supplement: Supplementary file 1 [file nutrients-14-01040-s001.zip › Nutrients_Gluten_Search_Strategy_FINAL.pdf]

Supplementary File S1. Search strategy

1. **Search strategy for EMBASE**

No.	Query
#1	celiac:ti,ab
#2	'celiac disease'/exp OR 'celiac disease':ti,ab
#3	'gluten enteropathy':ti,ab
#4	'gluten enteropathies':ti,ab
#5	'gluten sensitive enteropathy':ti,ab OR 'gluten sensitive enteropathies':ti,ab
#6	'celiac sprue':ti,ab
#7	'nontropical sprue':ti,ab OR sprue:ti,ab
#8	'gluten intolerance':ti,ab
#9	coeliac:ti,ab OR coeliaky:ti,ab OR coeliakya:ti,ab
#10	#1 OR #2 OR #3 OR #4 OR #5 OR #6 OR #7 OR #8 OR #9
#11	'breast feeding'/exp OR 'breast feeding':ti,ab OR 'breastfeeding':ti,ab OR 'breast milk'/exp OR 'breast milk':ti,ab OR 'human milk':ti,ab
#12	'breastfed':ti,ab OR 'breast fed':ti,ab OR 'maternal milk':ti,ab OR 'mother milk':ti,ab OR 'women milk':ti,ab
#13	#11 OR #12
#14	'gluten'/exp OR 'gluten':ti,ab OR 'glutens':ti,ab
#15	'secalin':ti,ab OR 'secalins':ti,ab OR 'hordein':ti,ab OR 'hordeins':ti,ab OR 'avenin'/exp OR avenin*:ti,ab OR 'legumin':ti,ab OR 'plant casein':ti,ab OR 'prolamin*':ti,ab OR 'prolamin'/exp
#16	'glutelin':ti,ab OR 'glutelins':ti,ab OR 'glutenin'/exp OR glutenin*:ti,ab
#17	#14 OR #15 OR #16
#18	(introduction:ti,ab OR introduce:ti,ab OR introduced:ti,ab OR introducing:ti,ab) AND (times:ti,ab OR time:ti,ab OR timing:ti,ab)
#19	amount*:ti,ab
#20	intake:ti,ab
#21	exposure:ti,ab
#22	quantity:ti,ab
#23	portion:ti,ab
#24	'weaning'/exp OR 'weaning':ti,ab OR 'weanings':ti,ab
	#25 'complementary feeding':ti,ab OR 'complementary feedings':ti,ab OR 'complementary feeding'/exp OR 'infant feeding'/de OR 'infant feeding':ti,ab
#26	'infant nutrition'/de OR 'infant nutrition':ti,ab OR 'baby food'/de OR 'baby food':ti,ab OR 'baby foods':ti,ab OR 'babyfood':ti,ab OR 'infant food':ti,ab OR 'infant foods':ti,ab OR 'early feeding':ti,ab OR 'solid food':ti,ab OR 'solid foods':ti,ab OR beikost:ti,ab OR beikosts:ti,ab
#27	infant:ti,ab AND (diet*:ti,ab OR 'dietary intake':ti,ab)
#28	'child'/exp OR 'child*':ti,ab OR 'childhood':ti,ab OR 'infant'/exp OR 'infant*':ti,ab OR 'infancy':ab,ti OR 'toddler*':ti,ab OR newborn*:ti,ab OR 'new born':ti,ab OR 'new borns':ti,ab OR 'newly born':ti,ab OR baby:ti,ab OR babies:ti,ab
#29	#18 OR #19 OR #20 OR #21 OR #22 OR #23 OR #24 OR #25 OR #26 OR #27
#30	#17 AND #29
#31	#13 OR #30

#32	#10 AND #28 AND #31

2. **Search strategy for MEDLINE (Pubmed)**

((((((((((((("Celiac Disease"[Mesh]) OR ("celiac disease"[Title/Abstract])) OR (celiac[Title/Abstract])) OR ("gluten enteropathy"[Title/Abstract])) OR ("gluten enteropathies"[Title/Abstract])) OR (("gluten sensitive enteropathy"[Title/Abstract]) OR ("gluten sensitive enteropathies"[Title/Abstract]))) OR ("celiac sprue"[Title/Abstract])) OR ("nontropical sprue"[Title/Abstract])) OR (sprue[Title/Abstract])) OR (((coeliac[Title/Abstract]) OR (coeliaky[Title/Abstract])) OR (coeliakya[Title/Abstract]))) OR ((("gee herter disease"[Title/Abstract]) OR (gee thaysen disease[Title/Abstract])) OR (gee heubner herter disease[Title/Abstract]))) OR (gluten intolerance[Title/Abstract])) AND (((((((("Glutens"[Mesh]) OR ((gluten[Title/Abstract]) OR (glutens[Title/Abstract]))) OR ((((secalin[Title/Abstract]) OR (secalins[Title/Abstract])) OR (hordein[Title/Abstract])) OR (hordeins[Title/Abstract]))) OR ((glutelin[Title/Abstract]) OR (glutelins[Title/Abstract]))) OR ("Prolamins"[Mesh])) OR (((((avenin*[Title/Abstract]) OR (legumin[Title/Abstract])) OR ("plant casein"[Title/Abstract])) OR (prolamin*[Title/Abstract])) OR (glutenin[Title/Abstract]))) AND ((((((((((((times[Title/Abstract]) OR (time[Title/Abstract])) OR (timing[Title/Abstract])) AND ((((introduction[Title/Abstract]) OR (introduce[Title/Abstract])) OR (introduced[Title/Abstract])) OR (introducing[Title/Abstract]))) OR (((((amount*[Title/Abstract]) OR (intake[Title/Abstract])) OR (exposure[Title/Abstract])) OR (quantity[Title/Abstract])) OR (portion[Title/Abstract]))) OR ("Weaning"[Mesh])) OR ((weaning[Title/Abstract]) OR (weanings[Title/Abstract]))) OR (("complementary feeding"[Title/Abstract]) OR ("complementary feedings"[Title/Abstract]))) OR ("infant feeding"[Title/Abstract])) OR ("infant nutrition"[Title/Abstract])) OR ((((((((("baby food"[Title/Abstract]) OR ("baby foods"[Title/Abstract])) OR ("babyfood"[Title/Abstract])) OR ("infant food"[Title/Abstract])) OR ("infant foods"[Title/Abstract])) OR ("early feeding"[Title/Abstract])) OR ("solid food"[Title/Abstract])) OR ("solid foods"[Title/Abstract])) OR (beikost[Title/Abstract])) OR (beikosts[Title/Abstract]))) OR ((infant[Title/Abstract]) AND ((diet*[Title/Abstract]) OR ("dietary intake"[Title/Abstract])))) OR ("Infant Food"[Mesh]))) OR ((((("breast feeding"[Title/Abstract]) OR (breastfeeding[Title/Abstract])) OR ("breast milk"[Title/Abstract])) OR ("human milk"[Title/Abstract])) OR (((((breastfed[Title/Abstract]) OR ("breast fed"[Title/Abstract])) OR ("maternal milk"[Title/Abstract])) OR ("mother milk"[Title/Abstract])) OR ("women milk"[Title/Abstract]))))) AND ((("Child"[Mesh]) OR "Infant"[Mesh]) OR (((((((((((child*[Title/Abstract]) OR (childhood[Title/Abstract])) OR (infant*[Title/Abstract])) OR (infancy[Title/Abstract])) OR (toddler*[Title/Abstract])) OR (newborn*[Title/Abstract])) OR ("new born"[Title/Abstract])) OR ("new borns"[Title/Abstract])) OR ("newly born"[Title/Abstract])) OR (baby[Title/Abstract])) OR (babies[Title/Abstract])))

3. **Search strategy for The Cochrane Central Register of Controlled Trials (CENTRAL, the Cochrane Library)**

ID	Search
#1	MeSH descriptor: [Celiac Disease] explode all trees
#2	("celiac disease"):ti,ab,kw
#3	(celiac):ti,ab,kw
#4	("gluten enteropathy"):ti,ab,kw OR ("gluten enteropathies"):ti,ab,kw

#5	("gluten-sensitive enteropathy"):ti,ab,kw OR ("gluten-sensitive enteropathies"):ti,ab,kw OR ("gluten sensitive enteropathy"):ti,ab,kw OR ("glutensensitive enteropathies"):ti,ab,kw
#6	("celiac sprue"):ti,ab,kw
#7	("nontropical sprue"):ti,ab,kw OR (sprue):ti,ab,kw
#8	(coeliac):ti,ab,kw OR (coeliaky):ti,ab,kw OR (coeliakya):ti,ab,kw
#9	("gluten intolerance"):ti,ab,kw
#10	{OR #1-#9}
#11	MeSH descriptor: [Breast Feeding] explode all trees
#12	("breast feeding"):ti,ab,kw OR (breastfeeding):ti,ab,kw AND ("breast milk"):ti,ab,kw AND ("human milk"):ti,ab,kw
#13	MeSH descriptor: [Milk, Human] explode all trees
#14	(breastfed):ti,ab,kw OR ("breast fed"):ti,ab,kw OR ("maternal milk"):ti,ab,kw OR ("mother milk"):ti,ab,kw OR ("women milk"):ti,ab,kw
#15	{OR #11-#14}
#16	MeSH descriptor: [Glutens] explode all trees
#17	(gluten):ti,ab,kw OR (glutens):ti,ab,kw
#18	(secalin):ti,ab,kw OR (secalins):ti,ab,kw OR (hordein):ti,ab,kw OR (hordeins):ti,ab,kw
#19	(glutelin):ti,ab,kw OR (glutelins):ti,ab,kw
#20	MeSH descriptor: [Prolamins] explode all trees
#21	(avenin*):ti,ab,kw OR (legumin):ti,ab,kw OR ("plant casein"):ti,ab,kw OR (prolamin*):ti,ab,kw
#22	(glutenin*):ti,ab,kw
#23	{OR #16-#22}
#24	(times):ti,ab,kw OR (time):ti,ab,kw OR (timing):ti,ab,kw
#25	(introduction):ti,ab,kw OR (introduce):ti,ab,kw OR (introduced):ti,ab,kw OR (introducing):ti,ab,kw
#26	#24 OR #25
#27	(amount*):ti,ab,kw OR (intake):ti,ab,kw OR (exposure):ti,ab,kw OR (quantity):ti,ab,kw OR (portion):ti,ab,kw
#28	MeSH descriptor: [Weaning] explode all trees
#29	(weaning):ti,ab,kw OR (weanings):ti,ab,kw
#30	("complementary feeding"):ti,ab,kw OR ("complementary feedings"):ti,ab,kw
#31	("infant feeding"):ti,ab,kw
#32	("infant nutrition"):ti,ab,kw OR ("infant food"):ti,ab,kw OR ("infant foods"):ti,ab,kw OR ("early feeding"):ti,ab,kw
#33	("baby food"):ti,ab,kw OR ("baby foods"):ti,ab,kw OR ("babyfood"):ti,ab,kw
#34	("solid food"):ti,ab,kw OR ("solid foods"):ti,ab,kw OR (beikost):ti,ab,kw OR (beikosts):ti,ab,kw
#35	(infant):ti,ab,kw
#36	(diet*):ti,ab,kw OR ("dietary intake"):ti,ab,kw
#37	#35 AND #36
#38	{OR #26-#34}
#39	#37 OR #38
#40	#23 AND #39
#41	#15 OR #40
#42	MeSH descriptor: [Child] explode all trees

#43	(child*):ti,ab,kw OR (childhood):ti,ab,kw OR (infant*):ti,ab,kw OR (infancy):ti,ab,kw OR (toddler*):ti,ab,kw
#44	(newborn*):ti,ab,kw OR ("new born"):ti,ab,kw OR ("new borns"):ti,ab,kw OR ("newly born"):ti,ab,kw OR (baby):ti,ab,kw
#45	(babies):ti,ab,kw
#46	MeSH descriptor: [Infant] explode all trees
#47	{OR #42-#46}
#48	#10 AND #41 AND #47
